# On the Treatment of Tremor

**Published:** 1873-03

**Authors:** Albert Eulenburg

**Affiliations:** Berlin


On the Treatment of Tremor—By Dr. Albert Eulenburg, of Berlin.—Muscular tremors of different grades of severity appear either as a phenomenon attached to many different morbid conditions, of which only a few—for instance diffuse sclerosis of the central nervous system—are anatomically well defined; while others are only symptomically and etiologically separated, as paralysis agitans, tremor senilis, alcoholicus, saturninus and mercurialis, or as an isolated symptom independent of any other discoverable fundamental morbid condition, as tremor simplex.

While its probable spinal origin is not yet fully established, it will not be unreasonable to suppose that the occurrence of all its forms is dependent upon the existence of the same common conditions, which, expressed in the most general terms, must consist partly in abnormal irritability, partly in abnormal irritation of certain motory nerve-spheres, either from the periphery or from the centers. All cases must etiologically belong prominently either to the one or to the other group; hence the possibility to attack them therapeutically upon a rational basis, even if the tremor should be only one of the prominent symptoms of another disease, must be admitted. Dr. Eulenburg has in a number of cases used remedies which have a decided paralyzing effect upon the central and peripheric portions of the motory nervous system—for instance curare—without any noticeable results. He has afterwards used those remedies which attack the peripheric sources from which motory nerves are excited, *by depressing reflex action or obstructing the conveyance of impressions within the tracts of sensitive nerves.* The principal representatives of this class are bromide of potasssium and arsenic. The first did not render him any material service. The latter was resorted to in consequence of the experiments of Sklarek,* who demonstrated that arsenious acid, and the salts it forms, with potash and soda, suspend the functional activity of that portion of the spinal cord which conveys sensations (impressions made upon nerves of sensation) while it does not interfere with the irritability of motory nerves.

Fowler's solution, (in the form of hypodermic injections, one part of the remedy to two parts of aqua distil. from 10 to 30 or more drops, representing, according to the German Pharmaco-

* Sklarek: Zur Physiologischen Wirkung der Arsenigen Säure. Archiv. von Reich[illegible] and du Bois Reymond, 1866, p. 481–493.

pœia, from one-fifth to three-tenths grain of the solution of arsenite of potassa,) have been used by him at one injection, with the most satisfactory results, and, notwithstanding the large doses, without producing toxical effects in any instance. The back of the neck, and neighborhood of the spinal column, have generally been selected for the application, and from fifteen to thirty injections, given once or twice a day, have generally been sufficient to obtain a very decided improvement. Dr. Eulenburg has treated seven cases of tremor in this manner, of which five were manifestly and decidedly benefitted.

It may not be out of place to add here that hypodermic injections of arsenite of potassa have been made by J. C. Lehman, of Copenhagen, in pernicious puerperal fevers, without effect; by von Graefe, in the algid stage of cholera; by Lewin, of Berlin, in psoriasis, with very marked benefit; and by Lewis Smith (*Med. Record*, 1872,), in cholera, with like results.—*Berliner Klinische Wochenschrift*, *No*. 46, 2872.

